# Selective Targeting of the Interconversion between Glucosylceramide and Ceramide by Scaffold Tailoring of Iminosugar Inhibitors

**DOI:** 10.3390/molecules24020354

**Published:** 2019-01-19

**Authors:** Cécile Baudoin-Dehoux, Tessa Castellan, Frédéric Rodriguez, Arnaud Rives, Fabien Stauffert, Virginie Garcia, Thierry Levade, Philippe Compain, Yves Génisson

**Affiliations:** 1 Laboratoire de Synthèse et Physico-Chimie de Molécules d’Intérêt Biologique (SPCMIB), Université Paul Sabatier-Toulouse III/ CNRS (UMR5068), 118 route de Narbonne, F-31062 Toulouse, France; castellan@chimie.ups-tlse.fr (T.C.); build@chimie.ups-tlse.fr (F.R.); rivesarnaud1@yahoo.fr (A.R.); 2 Laboratoire d’Innovation Moléculaire et Application (LIMA). Université de Strasbourg, Université de Haute-Alsace, CNRS (UMR 7042), Equipe Synthèse Organique et Molécules Bioactives (SYBIO), Ecole Européenne de Chimie, Polymères et Matériaux, 25 rue Becquerel, 67000 Strasbourg, France; fabien.stauffert@gmail.com; 3 Institut National de la Santé et de la Recherche Médicale (INSERM) UMR1037, Centre de Recherches en Cancérologie de Toulouse (CRCT), Université Paul Sabatier, Laboratoire de Biochimie Métabolique, Institut Fédératif de Biologie, CHU Purpan, F-31059 Toulouse, France; Virginie.Garcia@inserm.fr (V.G.); thierry.levade@inserm.fr (T.L.)

**Keywords:** iminosugars, glucocerebrosidase, pharmacological chaperones, glycosidase inhibitors, glucosylceramide synthase inhibitors, Gaucher disease, ligand docking

## Abstract

A series of simple *C*-alkyl pyrrolidines already known as cytotoxic inhibitors of ceramide glucosylation in melanoma cells can be converted into their corresponding 6-membered analogues by means of a simple ring expansion. This study illustrated how an isomerisation from iminosugar pyrrolidine toward piperidine could invert their targeting from glucosylceramide (GlcCer) formation toward GlcCer hydrolysis. Thus, we found that the 5-membered ring derivatives did not inhibit the hydrolysis reaction of GlcCer catalysed by lysosomal β-glucocerebrosidase (GBA). On the other hand, the ring-expanded *C*-alkyl piperidine isomers, non-cytotoxic and inactive regarding ceramide glucosylation, revealed to be potent inhibitors of GBA. A molecular docking study showed that the positions of the piperidine ring of the compound **6b** and its analogous 2-*O*-heptyl DIX **8** were similar to that of isofagomine. Furthermore, compound **6b** promoted mutant GBA enhancements over 3-fold equivalent to that of the related *O*-Hept DIX **8** belonging to one of the most potent iminosugar-based pharmacological chaperone series reported to date.

## 1. Introduction

**Scheme 1:**
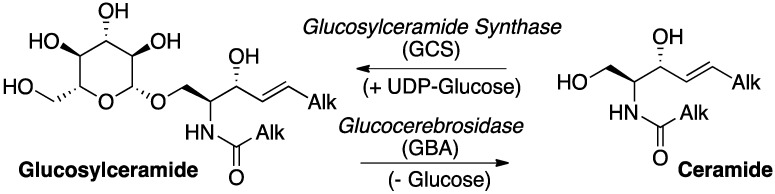
Metabolic interconversion between ceramide (Cer) and glucosylceramide (GlcCer).

Among the wide family of bioactive glycosphingolipids (GSLs), glucosylceramide (GlcCer) holds a central role [1]. First, because its biosynthesis from UDP-glucose and ceramide, catalysed in the endoplasmic reticulum (ER) by the enzyme glucosylceramide synthase (GCS), is the first committed biosynthetic step toward numerous complex GSLs, such as gangliosides, expressed at the surface of the cell membrane. Second, because its lysosomal hydrolysis under the action of the acid β-glucocerebrosidase (GBA) is the ultimate key catabolic step to regenerate the pivotal ceramide (Cer, Scheme 1).

The role of sphingolipids (SLs) as signalling molecules in diverse cell processes such as communication, adhesion, proliferation and differentiation further strengthens the importance of the metabolic manifold between GlcCer and Cer [2]. Indeed, whereas Cer is considered as a pro-apoptotic metabolite generated in response to different stress signals, GlcCer was proposed to act as a proliferative SL and its overproduction correlated to multidrug resistance of cancer cells [3]. On the other hand, genetic alterations leading to deficiency in lysosomal GlcCer degradation by GBA cause Gaucher disease (GD), a rare metabolic disorder belonging to the vast family of lysosomal storage diseases (LSDs) [4]. Furthermore, a correlation was recently established between mutations in the gene coding for GBA and the occurrence of α-synucleinopathies, such as the Parkinson disease, offering innovative therapeutic prospects [5,6,7,8].

Therefore, the search for pharmacological agents interfering with the balance between GlcCer and Cer is intensively pursued. Inhibiting the GCS-catalysed β-glucosylation of Cer was proposed as an approach to resensitize resistant cancer cell lines [9,10]. In addition, down-regulating the production of GlcCer through GCS inhibition was also developed as the substrate reduction therapy (SRT) for GD, the first oral small molecule therapy against LSD [11]. GBA inhibitors represent on the other hand a basis to develop pharmacological chaperones (PCs) able to restore the lysosomal activity of misfolded proteins, according to the enzyme enhancement therapy concept [12,13,14]. Since neuronopathic forms of GD are associated to severe symptoms, small molecules able to cross the blood-brain barrier are highly desirable [15,16].

**Figure 1:**
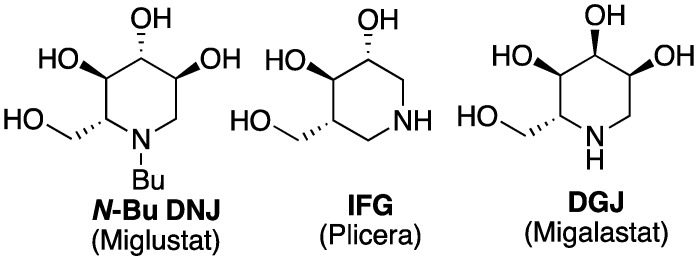
Examples of iminosugar therapeutic agents against LSDs.

In the field of LSDs, iminosugars represent an archetypical series of glycoside processing enzyme inhibitors [17], encompassing the emblematic 1-deoxynojirimycin (DNJ) glucose mimetics. The *N*-butyl derivative miglustat (Zavesca^TM^, *N*-butyl DNJ) (Figure 1), a moderately potent and selective oral GCS inhibitor, was successively approved for SRT as a second-line drug against non-neuronopathic type 1 GD in 2002 [18] and for neurological type C Niemann-Pick disease in 2009 [19,20]. It was additionally described as a PC restoring the activity of the mutated α-glucosidase involved in Pompe disease [21,22]. The homologous *N*-nonyl DNJ was on the other hand the first reported chemical chaperone for GBA [23,24]. The synthetic 1-*N*-iminosugar isofagomine (IFG), another well-studied PC for GBA [25,26], underwent a clinical trial against GD under the name of Plicera^TM^, eventually interrupted for lack of therapeutic efficiency. Finally, the isomeric galactose mimetic migalastat (deoxygalatonojirimycin, DGJ) was approved under the name Galafold^TM^ in 2016 against Fabry disease as a PC of the deficient α-galactosidase A [27].

**Figure 2:**
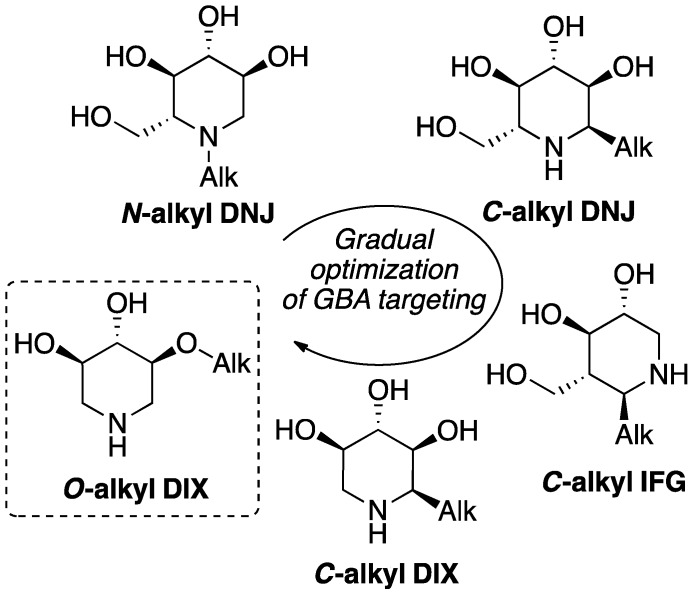
Structural evolution of GBA-targeting alkylated iminosugars.

Inspired by the seminal finding that a broad-spectrum glycosidase inhibitor could be addressed to Cer glycoside processing enzymes by incorporation of an *N*-alkyl substituent, structural variations toward more potent and selective analogues afforded a vast panel of *N*-, *O*- and *C*-alkyl 5-, 6 or 7-membered ring synthetic analogues, along with the reducing sp_2_ iminosugars. Specific targeting of GlcCer metabolism with plain iminosugar delineates interesting structure-activity trends (Figure 2). From the seminal *N*-alkyl DNJ series developed to address GBA inhibition, shifting the aliphatic chain around the heterocyclic core with gradual scaffold simplification led to 1-*C*-alkyl DNJ [28], 6-*C*-alkyl IFG [29,30], 1-*C*-alkyl [31,32,33,34,35] and finally 2-*O*-alkyl imino-d-xylitol (DIX) [36] as potent GBA inhibitors with significant PC activity at remarkably low concentrations. Although shorter chain lengths were considered, optimal selectivity and efficiency were usually observed with typical C7 to C9 linear aliphatic appendages.

**Scheme 2:**
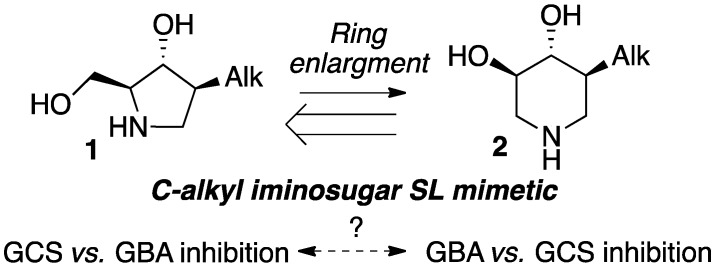
*C*-alkyl iminosugar sphingosine mimetics and opportunities thereof.

We previously disclosed a series of simple *C*-alkyl iminosugar-based sphingosine mimetics **1** as cytotoxic inhibitors of Cer glucosylation in melanoma cells (Scheme 2) [37,38]. Interestingly, such 5-membered iminosugars could be converted into the corresponding 6-membered analogues **2** by means of a simple chemical rearrangement [37,39,40]. Although the ring-expanded series **2** still shared structural similarity with sphingosine, their activity profiles were significantly altered. The resemblance of the *C*-alkylated 6-membered ring core **2** with 2-*O*-alkyl DIX GBA inhibitor scaffold (Figure 2) prompted us to further scrutinize the potential inhibitory activity of these iminosugars on either the formation or the hydrolysis of GlcCer. We report here our first efforts to assess the efficiency of both isomeric 5- and the 6-membered ring series in selectively targeting one of these two key metabolic steps.

## 2. Results and Discussion

### 2.1. Chemical Synthesis

**Scheme 3:**
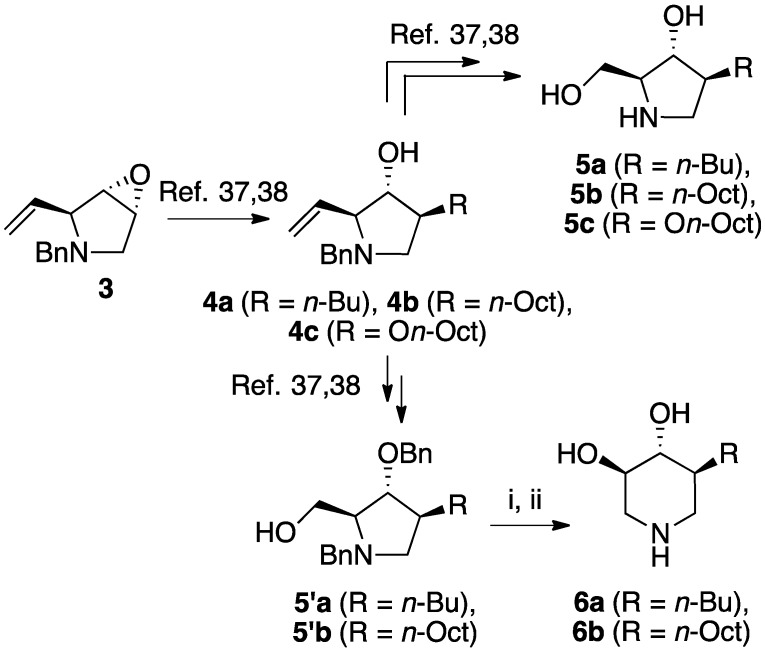
Asymmetric synthesis of *C*- and *O*-alkyl iminosugar sphingosine mimetics **5** and **6**. *Reagents and conditions:* The synthesis of all compounds, excepted **6b**, was previously described in ref. [37,38]. For the preparation of **6b** from **5′b**: (i) (CF_3_CO)_2_O (1.2 eq), 1,4-dioxane, 10 °C to RT, 1 h, then Et_3_N (4.0 eq), 90 °C, 90 h; (ii) 12 bars H_2_, Pd (OH)_2_/C (cat.), HCl 12N (cat.), MeOH, RT, 24 h (47% over two steps).

The enantiomerically enriched 5-membered ring *C*-alkyl sphingosine mimetics were readily obtained from the pivotal vinyl epoxypyrrolidine intermediate **3** (Scheme 3) [37,38]. Thanks to a regio- and stereoselective epoxide opening reaction, the *n*-butyl and *n*-octyl residues were efficiently incorporated to give **4a** and **4b**, respectively. Interestingly, the use of *n*-octanol as nucleophile also smoothly delivered the corresponding *O*-octyl analogue **4c**. Reductive ozonolysis of the vinyl appendage followed by hydrogenolysis of the *N*-benzyl group yielded the *C*- or *O*-alkyl 5-membered iminosugars **5a**–**c**. Alternatively, the secondary alcohol in the hydroxymethyl pyrrolidines **4a**,**b** was benzylated before oxidative cleavage of the olefin to deliver the *N*,*O*-diprotected intermediates **5′a**,**b**. The latter were engaged in the key ring-expansion reaction to deliver the isomeric *C*-alkyl 6-membered ring derivatives **6a**,**b** after catalytic hydrogenation.

**Scheme 4:**
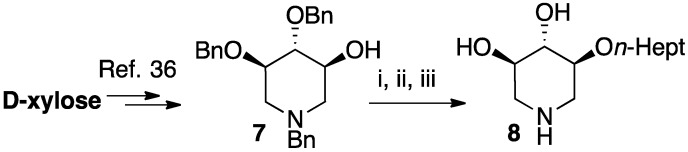
Chiral pool synthesis of the *O*-Hept DIX iminosugar of reference. *Reagents and conditions*: The synthesis of intermediate **7** was previously described in ref. 36. For the preparation of **8**: (i) NaH (2.3 eq), *n*-HeptBr (2.9 eq), DMF, RT, 16 h; (ii) H_2_, Pd(OH)_2_/C (cat.), *i*-PrOH/THF, RT, 20 h; (iii) Amberlite IRA400 (OH^−^), MeOH, 1 h.

The analogous 2-*O*-heptyl DIX **8**, bearing the same overall number of atom in the lipophilic appendage as the *C*-octyl piperidine **6b**, was introduced for the sake of comparison. The iminosugar **8** was synthesized from D-xylose following the synthetic sequence previously used to prepare the corresponding *O*-hexyl derivative (Scheme 4) [36]. 

*O*-alkylation of the protected DIX intermediate **7** followed by hydrogenolysis of all benzyl protecting groups delivered the expected 2-*O*-alkyl compound **8**.

### 2.2. Effect of Iminosugars on Cell Viability

Our initial studies delivered some key findings regarding the compounds’ cytotoxicity on B16 murine melanoma cells (Table 1). Compounds **5** were proposed to trigger cell death through accumulation of pro-apoptotic Cer [37,38]. While the impact of 5-membered ring iminosugar sphingosine mimetics **5** on cell viability was influenced by the alkyl chain length (*n*-Bu vs. *n*-Oct, i.e., **5a** vs. **5b**), at 25 μM, both *C*- and *O*-Oct derivatives **5b** and **5c** displayed an equivalent level of cytotoxicity. Comparison of *C*-Oct piperidine **6b**, resulting in 85% cell viability, with the parent *C*-Oct pyrrolidine **5b** clearly indicated however that the ring-expansion of the iminosugar framework almost completely abolished the impact on cell viability.

In order to complete this study in view of the evaluation of GBA inhibition, we assessed the impact at 30 μM of selected compounds on the viability of both control human fibroblasts and a cell line carrying the N370S mutation associated to GD. The observed trend was fully coherent with the previous set of data in murine melanoma cells. There again, both *C*- and *O*-Oct pyrrolidines **5b** and **5c** exerted a comparable cytotoxicity, leading to ca. 10–15% cell viability in both cells lines. In contrast, the *C*-Oct piperidine **6b** displayed a clearly different behaviour, resulting in 84 to 99% cell viability. For the sake of comparison, the analogous *O*-Hept DIX **8** was also evaluated, showing a slightly lower cytotoxicity. For the sake of comparison, the reference iminosugar IFG also showed marginal effect on cell viability (97% viability of Gaucher fibroblasts at 30 µM). These evaluations thus confirmed that switching from a 5- to a 6-membered ring iminosugar core markedly reduces the impact of these sphingosine mimetics on cell viability, not only in the *C*-alkyl, but also in the related *O*-alkyl series.

### 2.3. Effect of Iminosugars on GCS Inhibition

The observed structure-activity trends of *C*- and *O*-alkyl pyrrolidine and piperidine iminosugars **5** and **6** regarding GCS inhibition closely parallel that of their cytotoxicity (Table 2) [37,38]. The three cytotoxic pyrrolidines **5** displayed up to 47% GCS inhibition at 25 μM in melanoma cells, the most efficient compound being the *C*-Oct derivative **5b**. On the other hand, no detectable impact on GlcCer biosynthesis was observed upon treatment with the piperidine analogues **6a** or **6b**, indicating that the 5-membered ring scaffold of alkylated iminosugars **5** was required for their GCS inhibitory activity.

### 2.4. Effect of Iminosugars on GBA Inhibition

We further explored the biological profile of the iminosugar sphingosine mimetics with the evaluation of their in vitro GBA inhibitory potency using human recombinant GBA (Cerezyme^TM^, imiglucerase) at pH 5.4. The *O*-Hept DIX **8** was found to have an IC_50_ of inhibition of 4 nM, in agreement with the previously reported data for similar 2-*O*-alkyl DIX derivatives [36]. In sharp contrast, its *O*-alkyl pyrrolidine analogue **5c** displayed a 3 orders of magnitude higher IC_50_ (6 μM vs. 4 nM). Interestingly, the same trend was observed in the *C*-alkyl series. The previously identified 5-membered ring iminosugar GCS inhibitors **5a**,**b** exerted, at best, a modest GBA inhibition with IC_50_ values in the μM range. The *C*-octyl pyrrolidine **5b** displayed for instance an IC_50_ of GBA inhibition of 4.2 μM. This value was however equivalent to that of a related α-1*C*-octyl-imino-d-arabinitol (DAB) iminosugar-based inhibitor recently described [41,42]. On the other hand, the piperidine analogues **6a**,**b** displayed a two orders of magnitude higher potency. An IC_50_ of 11 nM was notably found for the most active *C*-Oct derivative **6b**, barely superior to that of *O*-Hept DIX **8**. The reference compound IFG gave an IC_50_ of GBA inhibition of 58 nM in agreement with reported data [43]. The dramatic impact of the iminosugar scaffold of the sphingosine mimetics **5** and **6** on their biological profile was thus observed to also operate with GBA inhibition. It overall afforded a clear-cut orthogonality between GCS inhibition by 5-membered ring alkylated iminosugars **5** and GBA inhibition by the corresponding ring-expended series **6**.

### 2.5. Molecular Docking

**Figure 3:**
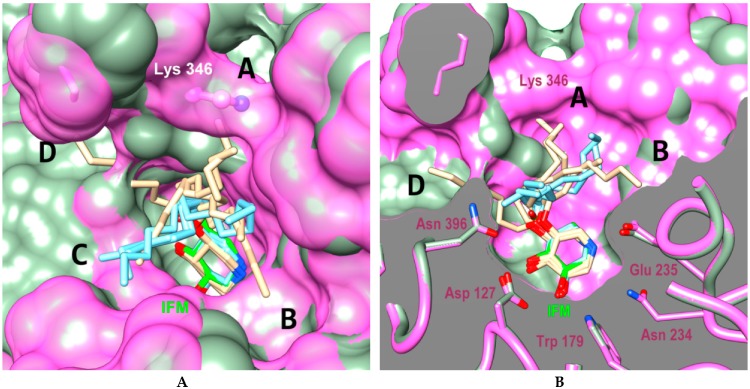
Combination of best docked poses for compound **8** using protocols P1-OPT (brown) and P2-GPU (cyan) in 2NSX (chain B, light green) and 2WCG (chain A, pink surface) binding cavity (**A**: surface view, **B**: clipped view). The PDB ligand IFM (IFG) is shown in green in the bottom of active site and LYS346 residue of 2WCG is highlighted. Letters **A** to **D** reffer to the channels explored by the lipophilic chains.

**Figure 4:**
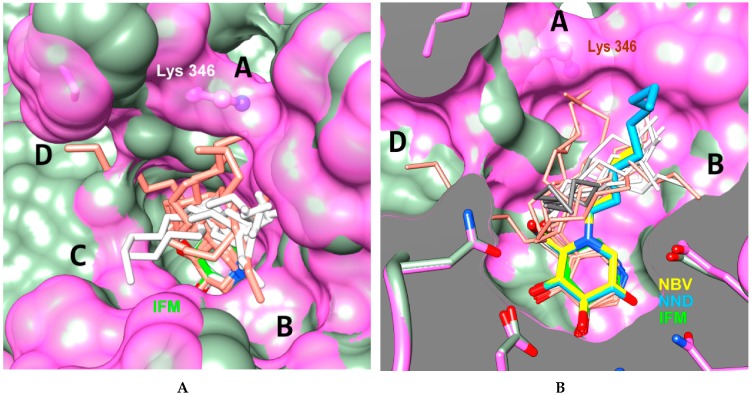
Combination of best docked poses (sticks, thin sticks) for compound **6b** using protocols P1-OPT (salmon) and P2-GPU (white or gray) in 2NSX (chain B, light green) and 2WCG (chain A, pink surface) binding cavity (**A**: surface view, **B**: clipped view). The PDB IFM ligand (IFG) (green) of structure 2NSX is shown in the bottom of active site and LYS346 residue of 2WCGa is highlighted. The PDB ligands NBV (*N*-butyl DNJ) (yellow, structure 2V3D, chain A), NND (*N*-nonyl DNJ) (blue, structure 2V3E, chain A) and aliphatic chains of **6b** that fluctuate in C channel (gray, other poses in white) are shown in Figure 4B. Letters **A** to **D** reffer to the channels explored by the lipophilic chains.

Prompted by the high inhibitory potency of piperidines **6b** and **8**, we decided to gain insight into their yet undocumented protein binding by means of molecular docking. Due to their structural similarity with other iminosugar-based competitive GBA inhibitors, we hypothesized that **6b** and **8** did also bind to the enzyme active site. The crystal structures of GBA in complex with different ligands (PDB structure id: 2NSX, ligand id: IFM and PDB structure id: 2WCG, ligand id: MT5) were used for the docking analysis. The Figure 3 shows combined docking results obtained for compound **8** after calculation using the two independent protocols P1-OPT (brown poses) and P2-GPU (cyan poses) detailed in the experimental part. The protocol P1-OPT (protein flexibility during the docking) allowed a greater fluctuation of the aliphatic chains whereas the protocol P2 (P2-GPU) was based on a rigid docking at protein level. Overall, the calculated placement of the piperidinic core of **8** was fully conform with that of IFG/IFM in the co-crystal structure with the protein. A similar hydrogen bond network was thus found to surround the iminosugar framework as shown in Figure 3B (interactions of ASP127, TRP381, TRP179 with alcohol functions and interactions of GLU340, GLU235 with the protonated nitrogen atom). Regarding the highest ranked combined (P1-OPT and P1-GPU) poses obtained for compound **8**, the lipophilic chains were found to explore four distinct channels (marked as A–D, Figure 3 and Figure 4) surrounding the binding cavity, particularly in the case of P1-OPT protocol. In addition, in structure 2NSX, P1-OPT protocol allows displacement of VAL398 and ASN396 to open channel D in the best pose and population of the channel A left open by LYS346 for other poses. 

The same type of iminosugar scaffold placement and aliphatic chain fluctuations were recorded with compound **6b** (Figure 4). We further performed a comparison with the crystallographic binding of *N*-alkyl-DNJ to GBA (PDB structure id: 2V3D, ligand id: NBV and PDB structure id: 2V3E, ligand id: NND) [44]. As reported, the positioning of *N*-nonyl DNJ/NND and IFG/IFM in the protein active site is mainly directed by the three hydroxyl groups that display strictly conserved positions among the different co-crystal structures, whereas the nitrogen location is shifted [44]. The secondary amine in the 1-*N*-iminosugar IFG/IFM is indeed structurally analogous to the anomeric carbon of the glucose residue in GlcCer. On the other hand, the tertiary amine of *N*-nonyl DNJ/NND mimics the glucopyranoside endocyclic oxygen. As the branching of the alkyl chain on the iminosugar scaffold of compounds **6b** and **8** is shifted by two carbon units relatively to *N*-nonyl DNJ/NND, the modelled IFG/IFM-type binding of the piperidinic core induces a somewhat disperse aliphatic chain cavity occupancy. The subsequent population of the four surrounding channels A-D was observed mainly with P1-OPT protocol whereas we noticed a trend for the alkyl chains of **8** and **6b** to match with the extremity of that of *N*-nonyl DNJ/NND with P2-GPU protocol.

### 2.6. GBA Pharmacological Chaperone Activity

Encouraged by the significant GBA inhibitory potency of the *C*-Oct 6-membered ring iminosugar **6b**, we further studied the potential of this alkylated piperidine to act as a PC able to enhance the GBA activity in GD patient cells.

#### 2.6.1. Preliminary In Vitro Studies

**Figure 5:**
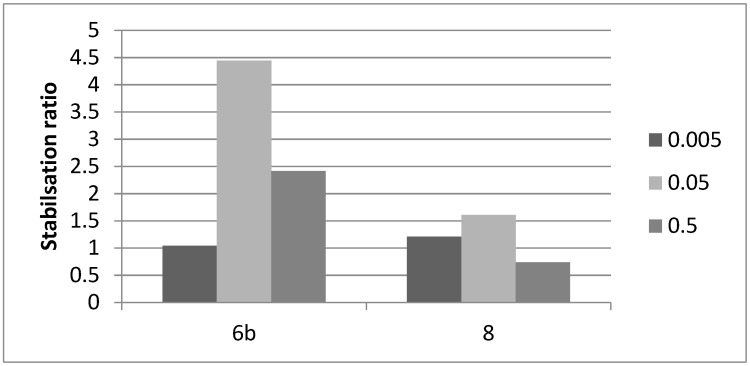
Ability of compounds **6b** and **8** to stabilize GBA. Enzyme activity was tested in the absence or presence of the inhibitor at 0.5, 0.05 and 0.005 µM. Data are expressed as the stabilization ratio (the ratio of relative enzymatic activities measured in the presence of inhibitor *vs* control) of after thermal denaturation (48 °C) for 60 min at the indicated inhibitor concentrations (µM).

We first assessed the stabilization of recombinant GBA under thermal denaturation conditions as described previously [23]. We investigated the effect of the most potent GBA inhibitors **6b** and **8** on the protein stabilization at 48 °C. The GBA was incubated in 100 mM McIlvaine buffer (pH 5.4) containing 0.25% sodium taurocholate and 0.1% Triton X-100 for 60 min and the remaining enzyme activity was determined with 4-methylumbellifery-β-d-glucopyranoside as substrate. The enzyme activity was lost within 60 min under incubation without test samples (9% activity remained at 60 min). In contrast, the piperidines **6b** and **8** exhibited stabilization ratios of 4.4 and 1.6 respectively at 0.05 µM (Figure 5). These results are in accordance with stabilization ratio obtained for 2-*O*-hexyl-DIX [36]. At such a low concentration, no stabilizing effect was observed at all with the reference iminosugar IFG. The reduced apparent stabilisation at 0.5 µM may reflect an undesired inhibitory effect of the compounds at higher concentration.

#### 2.6.2. Enhancement of GBA Activity in GD Patient Cells

In order to confirm the preliminary in vitro PC activity observed for the *C*-octyl iminosugar **6b**, we further tested the PC activity of this set of GBA inhibitors in human fibroblasts. Cell lines derived from homozygous GD patients bearing the most prevalent N370S mutation, commonly associated to the non-neuronopathic type 1 of the GD, were selected. 

**Figure 6:**
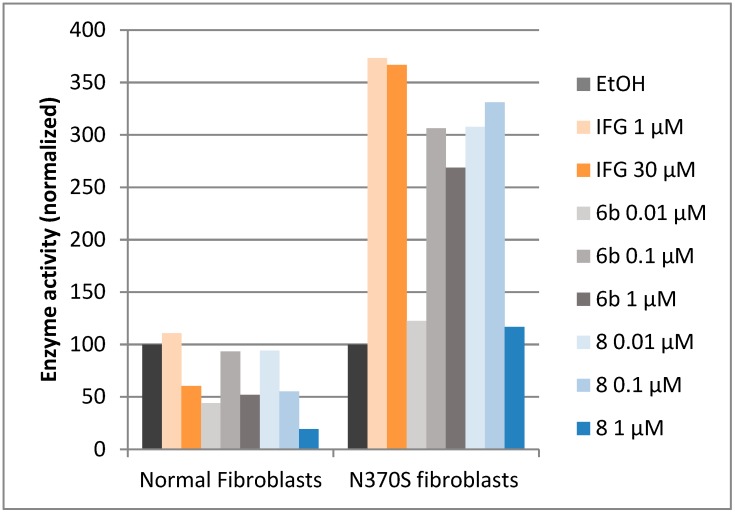
Effect of IFG, **6b** and **8** on lysosomal GBA activity in N370S fibroblasts (Gaucher disease type 1) after 72 h. In the absence of added compounds, the GBA activity in N370S fibroblasts averaged 6.4 nmol/h·mg while that in normal fibroblasts it approximated 43.0 nmol/h·mg.

Treatment with 1 or 30 μM of the reference PC molecule IFG led, after 3 days of incubation, to a 3.5-fold enhancement of GBA activity compared to untreated cells, in concordance with previous reports (Figure 6) [25,26]. At such high concentration, the *O*-Hept DIX 8 gave almost no effect, likely because of a counterproductive enzyme inhibition. Reducing the concentration in DIX 8 down 100 or 10 nM resulted in a more than 3-fold increase of enzyme activity. Such a low-concentration effect is concordant with the results previously reported at the same concentration in N370S GD fibroblasts for the corresponding *O*-hexyl analogue [36]. To our satisfaction, the *C*-Oct piperidine **6b** also behaved as a potent PC, giving an equivalent 3-fold enhancement of GBA activity at the optimal concentration of 100 nM. While 10 nM in **6b** gave almost no effect, enhancing the concentration to 1 μM led to a slightly lower increase in GBA activity, possibly due to its inhibitory effect. It is worth mentioning that examples of small chaperone molecules enhancing GBA activity at sub-micromolar concentrations are limited [45].

## 3. Discussion

We compared the relative impact of two isomeric series of alkylated iminosugar SL mimetics on the metabolism of GlcCer. The 5-membered ring derivatives, reported as cytotoxic inhibitors of the GCS-catalysed biosynthesis of GlcCer in murine melanoma cells, did not inhibit the reverse hydrolysis reaction catalysed by GBA. On the other hand, the ring-expended *C*-alkyl piperidine isomers, non-cytotoxic and inactive regarding the Cer glucosylation, revealed to be potent inhibitors of the GlcCer degradation. This study illustrated how a subtle isomerisation of the iminosugar pyrrolidine or piperidine scaffold could lead to a selective targeting of the GlcCer formation or hydrolysis. Moreover, the *C*-Oct derivative **6b** displayed an IC_50_ of GBA inhibition of 11 nM, comparable to that of *O*-Hept DIX **8**. Two series of iminosugars obtained by the same divergent synthetic strategy are thus able to display opposite activity on the metabolic interconversion between Cer and GlcCer, by either inhibiting GlcCer degradation (6-membered derivatives) or by inhibiting GlcCer formation (5-membered derivatives). To gain insight into the protein binding of these potent GBA inhibitors, molecular docking study was performed. The positions of the piperidine ring of the compound **6b** and its analogous 2-*O*-heptyl DIX **8** in the GBA active site were similar to that of isofagomine whereas the alkyl chain may occupy four different channels. In vitro experiments on homozygous N370S GD fibroblasts further revealed that this *C*-octyl iminosugar also acted as a PC for GBA. Compound **6b** used at 100 nM was able to induce a 3-fold increase in GBA activity after 72 h of incubation, an effect equivalent to that of the related *O*-Hept DIX **8** belonging to one of the most potent iminosugar-based N370S GBA PC series reported to date. Thanks to the flexible enantioselective synthetic route to **6b**, this finding thus opens wide prospects for the exploration of the structure-activity relationships in this series.

## 4. Materials and Methods

### 4.1. General Methods

All reactions requiring anhydrous conditions were carried out under nitrogen. Analytical thin-layer chromatography (TLC) was performed by using silica gel 60F254 precoated plates (Merck St. Louis, MO, USA). Chromatograms were observed under UV light and/or were visualized by heating plates that had been dipped in 10% phosphomolybdic acid in ethanol (Sigma-Aldrich, St. louis, MO, USA) or Dragendorff reagent. Flash column chromatography was carried out with 35–70 µm silica gel (Merck, St. Louis, MO, USA)). NMR spectroscopic data were obtained on an Advance 300 spectrometer (Bruker, Billerica, MA, USA) operating at 300 MHz or 400 MHz for ^1^H-NMR analysis, and 75 MHz and 100 MHz, respectively, for ^13^C-NMR analysis. Chemical shifts are quoted in parts per million (ppm) relative to the residual solvent peak and coupling constants are given in hertz. For convenience, assignments of chemical shifts are described according to the numbering drawn in Supplementary Materials. Infrared (IR) spectra were recorded with a FT-IR 1725X spectrometer (Perkin-Elmer, Waltham, MA, USA). High-resolution mass spectra (HRMS) were recorded with a MAT 95 XL spectrometer (ThermoFisher, Waltham, MA, USA). Optical rotations were measured with P-2000 polarimeter (JASCO, Tokyo, Japan). 

### 4.2. Synthesis of Compounds ***6b*** and ***8***

*(3R,4R,5S)-5-Octylpiperidine-3,4-diol* (**6b**). To a solution of the prolinol **5′b** (57.2 mg, 0.14 mmol) in anhydrous 1,4-dioxane (1.4 mL) at 10 °C under an inert atmosphere was added trifluoroacetic anhydride (23.4 µL, 0.17 mmol, 1.20 eq). The mixture was then stirred for 1 h at room temperature before Et_3_N (78 µL, 4 eq) was added. After 15 min, the mixture was heated to 90 °C for 4 days and then allowed to cool before a 2.5 M aqueous NaOH solution (0.5 mL, 9 equiv.) was added. After 1 h, brine was added and the aqueous layer was extracted with CH_2_Cl_2_. The combined organic phases were washed with brine, dried with Na_2_SO_4_, filtered and evaporated to dryness. The crude product was purified by flash column chromatography on silica gel (petroleum ether (PE)/EtOAc 70:30 to 60:40, 0.8% NH_4_OH) to give the desired compound as a white solid (35.9 mg). Pd(OH)_2_/C (10 mg) and 12 N HCl aqueous solution (1 drop) were successively added to this solid dissolved in MeOH (2 mL). The flask was purged with N_2_ and then loaded with H_2_ (10–12 bars). The mixture was stirred at room temperature until disappearance of the starting material (24 h). The catalyst was then removed by filtration trough Celite and the filtrate evaporated to dryness. The residue was taken up in 2:1 MeOH/water and Dowex 50WX8-200 ion-exchange resin (Sigma-Aldrich, St. louis, MO, USA) was added. After being stirred for 1 h, the resin was successively filtered and washed with water and methanol. A 3 M ammonium hydroxide solution was then added and the suspension was stirred for 1 h before being filtered and rinsed with a 3 M ammonium hydroxide solution (500 mL/mmol). The resulting solution was evaporated to dryness under pressure to give **6b** as a white solid (15 mg, 47% over 2 steps). ${\left[\alpha \right]}_{D}^{25}$ +23.5 (c 1.0; MeOH). IR (neat) 3277 (OH), 1049 (C–O) cm^−1^. ^1^H-NMR (300 MHz, CD_3_OD) δ 3.35 (ddd, *J* = 10.7, 8.7, 5.0 Hz, 1H, H-4), 3.07 (ddd, *J* = 12.1 Hz, 5.0 Hz, 1.5 Hz, 1H, H-5′), 2.99–3.05 (m, 1H, H-1′), 2.97 (dd, *J* = 10.1, 8.7 Hz, 1H, H-3), 2.34 (dd, *J* = 12.1 Hz, 10.7 Hz, 1H, H-5), 2.18 (t, *J* = 12.4 Hz, 1H, H-1), 1.76–1.87 (m, 1H, H-6′), 1.11–1.39 (m, 13 H, CH_2_-12, CH_2_-12 CH_2_-11, CH_2_-10, CH_2_-9, CH_2_-8, H6), 0.90 (t, *J* = 6.9 Hz, 3H, CH_3_-H13). ^13^C-NMR (75 MHz, CD_3_OD) δ 79.3 (C-3), 74.1 (C-4), 52.2 (C-5), 50.6 (C-1), 44.5 (C-2), 29.5 (C-7), 29.4 (C-9), 29.1 (C-10), 28.0 (C-11), 24.2(C-12), 14.5 (C-13). HRMS (IC) calculated for C_13_H_28_NO_2_: 230.2120 (M + H^+^), found 230.2119.

*(3R,4S,5S)-(Heptyloxy)piperidine-3,4-diol* (**8**). To a solution of compound **7** (35 mg, 0.0867 mmol) in dry DMF (1.2 mL) was added NaH (2.3 eq., 8 mg, 0.20 mmol, 60% on mineral oil) at 0 °C. The mixture was stirred at room temperature for 30 min then 1-bromoheptane (2.9 eq., 0.04 mL, 0.2546 mmol) was added and the reaction mixture was stirred at room temperature for 16 h. The reaction was quenched by slow addition of water, and the reaction mixture was extracted twice with AcOEt. The combined organic extracts were washed with sat. NaHCO_3_ and brine, dried over MgSO_4_, filtered and concentrated under reduced pressure. The crude residue was purified by flash column chromatography on silica gel (EtOAc/PE 6:94) to afford the desired compound (40 mg, 92%) as a colorless oil. The latter (38 mg, 0.0757 mmol) was dissolved in *i*PrOH (1 mL) and THF (1 mL), then 1 M HCl aqueous solution (0.6 mL) and Pd(OH)_2_/C (0.2 eq., 16 mg, 0.0114 mmol, 20% Pd on carbon, nominally 50% water) were added. The flask was evacuated and backfilled with argon (four cycles) and then evacuated and backfilled with H_2_ (four cycles). The reaction mixture was stirred under an atmosphere of H_2_ (1 atm) at room temperature for 20 h. The reaction mixture was then filtered through a pad of Celite (previously rinsed with at least 250 mL of 1M HCl aqueous solution) and rinsed with MeOH. The filtrate was concentrated under reduced pressure. The residue was dissolved in MeOH then ion exchange resin Amberlite^®^ IRA400 (OH^−^) (Sigma-Aldrich, St. louis, MO, USA) was added and the suspension was rotated at room temperature for 1 h. The resin was filtered, rinsed with MeOH and the filtrate was concentrated under reduced pressure. The crude residue was purified by flash column chromatography on silica gel (CH_2_Cl_2_/MeOH 80:20) to afford **8** (16 mg, 91%) as a white solid. ${\left[\alpha \right]}_{D}^{20}$ = +18 (*c* 1.0, MeOH). IR (neat) 3286 (O–H, N–H), 2924 cm^−1^ (C-H). ^1^H-NMR (CD_3_OD, 400 MHz) δ 0.90 (t, *J* = 6.8 Hz, 3H; CH_3_-12), 1.26–1.40 (m, 8H; CH_2_-8, CH_2_-9, CH_2_-10 and CH_2_-11), 1.53–1.62 (m, 2H; CH_2_-7), 2.26–2.34 (m, 1H; H^A^-1), 2.36 (dd, *J* = 12.4, 10.2 Hz, 1H; H^A^-5), 3.02 (ddd, *J* = 12.4, 4.9 and 1.0 Hz, 1H; H^B^-5), 3.10–3.19 (m, 2H; H^B^-1 and H-2), 3.24 (t, *J* = 8.4 Hz, 1H; H-3), 3.39 (ddd, *J* = 10.2, 8.4 and 4.9 Hz, 1H; H-4), 3.59 ppm (t, *J* = 6.7 Hz, 2H; CH_2_-6). ^13^C-NMR (CD_3_OD, 100 MHz) δ 14.4 (C-17), 23.7 (C-11), 27.1, 30.3, 31.2, 33.0 (C-7, C-8, C-9, C-10), 49.0 (C-1), 51.4 (C-5), 71.9 (C-6), 72.7 (C-4), 79.0 (C-3), 80.8 ppm (C-2). HRMS (ESI) calculated for C_12_H_26_NO_3_: 232.1907 [M + H]^+^, found 232.1899.

### 4.3. Cellular Inhibition Assays against Glucosylceramide Synthase

Evaluation of GCS inhibition was performed as previously reported [37,38]. The in vivo metabolism of sphingolipids was monitored by incubating intact cells for 4 h with 5 μM NBD-C6-ceramide, delivered by ethanolic injection. Cells were then harvested, lysed in a small volume of water (0.5 mL) and lipids were extracted by adding 2.5 mL of chloroform/methanol (2:1, *v*/*v*). After centrifugation (1000× *g*, 5 min), the lower phases were dried under nitrogen and subjected to TLC by using chloroform/methanol/32% ammonia (70:30:5, *v*/*v*) as the mobile phase. NBD-C6-GlcCer was eluted from the silica and quantified spectrofluorometrically (λex = 470 nm and λem = 530 nm) by comparison with known amounts of NBD-lipid.

### 4.4. Inhibition Assays against Recombinant β-glucocerebrosidase.

Evaluation of GBA inhibition was performed as previously reported [46]. The inhibitory activity toward β-glucocerebrosidase was measured with Cerezyme^TM^ (Sanofi Genzyme, Cambridge, MA, USA) as the enzyme source. Enzyme solutions (25 μL from a stock solution containing 0.125 mg/mL) in the presence of 0.25% (*w*/*v*) sodium taurocholate and 0.1% (*v*/*v*) Triton X-100 in Mcllvaine buffer (100 mM sodium citrate and 200 mM sodium phosphate buffer, pH 5.4) were incubated at 37 °C without (control) or with inhibitor at a final volume of 90 μL for 30 min. After addition of 10 μL 4-methylumbelliferyl-β-d-glucopyranoside (4 mM, Mcllvaine buffer pH 5.4), the samples were incubated at 37 °C for 10 min. Enzymatic reactions were stopped by the addition of aliquots (150 μL) of glycine/NaOH buffer (100 mM, pH 10.6). The amount of 4-methylumbelliferone formed was determined with a fluorimeter at 355 nm (excitation) and 460 nm (emission). IC_50_ values were determined by plotting percent activity versus log [I], using at least five different inhibitor concentrations.

### 4.5. Molecular Docking

Molecular graphics were performed with the Chimera package (University of California, San Francisco, CA, USA) [47]. Chimera is developed by the Ressource for Biocomputing, Visualization, and Informatics at the University of California, San Francisco (supported by the NIGMS P41-GM103311). The protein structures were downloaded from the RCSB Protein Database [48] and were structurally aligned against the structure 1OGS [49] (chain A) set as reference and using UCSF Chimera/Matchmaker [50] program. The protein structures, in this reference space, were prepared (structure checks, rotamers, hydrogenation, splitting of chains) using Biovia (www.3dsbiovia.com) Discovery Studio Visualizer 2016 (DSV) (Biovia, San Diego, CA, USA) and UCSF Chimera. The new compounds were sketched using ChemAxon Marvin 16, (Chemaxon, Budapest, Hungary). All ligands (extracted from protein structures or new) were checked (hybridization, hydrogenation, some geometry optimizations, 3D sketching) and were merged in SDF libraries using DSV. The structurally aligned GBA structures were clustered using a combined fluctuation analysis of: (i) structural motifs (defined by residues 346–349, 393–399, 312–319); (ii) residues (TYR313, PHE397, PHE347, LYS346) and (iii) surface of binding cavity. This gives four clusters for relatively closed structures and three clusters (A, B, C) for opened structures. The structures 2NSX (chain B, 2NSXb), 2XWD (chain A, 2XWDa) were found representatives of cluster A. The structures 2V3Da, 2WCGa, 3GFXb were found representatives of cluster B. The clusters A and B show a net fluctuation of LYS346 closing (cluster B) a surface channel near binding cavity. The active site of cluster C is less opened (overlying position of TYR313) than the one of clusters A-B. According to these analyses, the molecular docking protocols were applied on clusters A and B. Molecular modeling studies [51] were carried with Molegro Virtual Docker 6 software (www.clcbio.com) using the B chain of structure 2NSX [52] and the A chain of 2WCG [53] as targets. A search space volume of 15 Å radius centered in the binding pocket was used. According to structural study, 23 residues were defined as flexible (for 2NSXb and 2WCGa) during the docking: ALA238, ASN234, ASN396, ASP127, ASP283, CYS342, GLN247, GLN284, GLU235, GLU340, LEU241, LEU314, LYS346, PHE128, PHE246, PHE347, PHE397, SER345, TRP179, TRP381, TYR244, TYR313 and VAL398. The ligands were set flexible during the docking, and two different docking protocols (searching algorithms, flexibility) were used. The protocol P1 (P1-OPT) is based on flexible docking at protein level (softened potentials during docking phase) and Moldock optimizer used as searching algorithm. Docking process uses 10,000 iteration steps, other parameters let as default, 20 independent runs) for searches. Final minimization was set using 4000 steps for lateral chains and protein backbone; other parameters were let with default values. No water molecules (entropy penalty) were taken in account in the study. MolDock and Rerank [54] scores were calculated post-docking and post-minimization. The protocol P2 (P2-GPU) is based on rigid docking at protein level and a GPU (Nvidia Tesla CUDA hardware, www.nvidia.com) screening algorithm. Docking process uses the MolDock function (Moldock [grid] with a resolution of 0.2 Å) for scoring and CUDA optimizer (MVD, 10,000 iteration steps, other parameters let as default, 40 independent runs) for searches. Post-docking, all the poses were submitted to a short energy minimization/H-bonds optimization and re-ranked using MolDock and Rerank scoring systems. Using aligned protein structures in the same reference space, the conformations and positions of co-crystallized GBA ligands (NBV, IFM, NND, BTB, MT5, LGS, 3RI, 3RK) show clearly a lot of similarities. This is particularly the case for iminosugar compounds that share similar positions of primary or secondary OH groups. Using these structural data, pharmacophoric models (templates) were defined on a basis of structurally aligned atoms of same chemical features (such as hydrogen donor or acceptor) included in spheres centered on the mean position of the corresponding co-crystallized ligand atoms. The templates are used to define a similarity score contribution that is distance dependent and defined by a mathematical function (Gaussian type, parameters are radius = 1.8 Å and strength). Four docking templates were defined: (i) a steric group of 10 atoms (strength = 0.5) and three groups (strength = 1) for (ii) hydrogen donors (four atoms); (iii) a hydrogen acceptors (five atoms) and (iv) a ring group (five atoms). The cyclic nitrogen atoms of ligands are not included in the previous templates. Using these definitions, templates scoring (similarity score) were used in calculations in the case of the two protocols P1-OPT and P2-GPU, with a grid resolution of 0.3 Å and a global strength of 1000. These calculation parameters do not induce too strong constraints (i.e., poses outside binding cavity can be easily obtained) but favor obtaining of conforming poses. The same post-docking filtering protocol was also used for the two protocols. The clustering parameters (RMSD threshold 1.5 Å, other parameters let as default) were used. The poses issued from the calculations (20 poses for P1, 40 for P2) were carefully inspected for (i) strongness of scores (ability for a given pose to be, at once, the best value for each score) and for (ii) conformity (ability to reproduce the hydrogen bond network found with co-crystallized ligands found in PDB structures). The two protocols were applied to co-crystallized ligands in order to check the reproduction of X-ray structures. Typical best RSMD values between predicted and crystallographic conformations of a same ligand were found lower to 0.7 Å for the cyclic core. The fluctuation of hydrophobic chains can increase the values of RMSD while maintaining conformity of the cyclic cores. These protocols are able to reproduce with a good accuracy the typical hydrogen bond network (with residues ASN234, ASN396, ASP127, CYS342, GLU235, GLU340, SER345, TRP179, TRP381). The protocol P2 (receptor flexibility disabled) gives generally a subset of best poses issued from protocol P1 (receptor flexibility enabled) showing that the sampling of solution’s space is real. When a given pose was found to be strong (the better for MolDock and Rerank scores) we noticed that this pose was very often conform.

### 4.6. Thermal Stabilization Assay Using Recombinant β-Glucocerebrosidase

The enzyme assay methods were similar to those previously reported [23]. Commercial Cerezyme aliquots (38 µL, 2 mg/mL in MacIlvaine Buffer pH = 7.0) were incubated with three different concentrations of chemical chaperone at 48 °C. Subsequently, 220 µL of 0.1 M acetate-phosphate buffer (pH 5.0) and 30 µL of 4-methylumbelliferyl-β-d-glucopyranoside (4 mM, 0.1% Triton X-100 and 0.2% sodium taurocholate in McIlvaine buffer, pH 5.4) were added and incubated for additional 10 min at 37 °C. Then, 300 µL of glycine/NaOH buffer (100 mM, pH 10.6) were added. The amount of 4-methylumbelliferone formed was determined with a fluorimeter at 355 nm (excitation) and 460 nm (emission).

### 4.7. Culture and Cell Viability Assays

Cultured primary skin fibroblasts from control individuals and patients affected with Gaucher disease (carrying the N370S mutation) were obtained from the Laboratoire de Biochimie Métabolique, CRB, IFB, CHU Toulouse, France and CBC Biotec biobank BB-0033-00046 (3809 F01 and 1541 F02 cell lines), Hospices Civils de Lyon, France. Cells were immortalized after tranfection with a plasmid encoding the SV40 large T antigen. Cells were routinely cultured in DMEM medium supplemented with 10% inactivated foetal calf serum. After incubation for 72 h in the presence of the chemical compounds or vehicle (ethanol) only, cell viability was evaluated using the MTT test as reported earlier [55]. Briefly, cells were plated in 24-well plates (30,000 cells/well) and at 50% confluency cells were incubated with fresh medium supplemented with varying concentrations of compounds. After 72 h, 500 μg of MTT was added to each well. After 2 h of incubation at 37 °C, supernatants were discarded, and the remaining material was dissolved by 1 mL of DMSO. After 30 min, absorbance was measured at 560 nm by using a microplate reader. Background values were subtracted from all other values and viability was expressed as percentage compared with untreated controls.

### 4.8. Assay of β-glucocerebrosidase Activity

Following incubation of the fibroblasts for 72 h in the presence or absence with the chemical compounds, cells were harvested and pelleted. Cell lysates were prepared in 0.2% Triton X100 by brief sonication. β-glucocerebrosidase enzyme activity was determined on cell lysates in sodium acetate buffer pH 5.6 using 4-methylumbelliferyl-β-d-glucopyranoside (Sigma-Aldrich, St. louis, MO, USA) as substrate [55].

## Figures and Tables

**Table 1:** Effect of iminosugars on cell viability.

Cell Viability ^1^	Murine Melanoma ^2^	Human Fibroblast ^3^	Gaucher Fibroblast ^4^
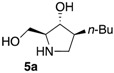	85%	ND ^5^	ND
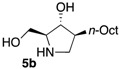	14%	12.5%	14%
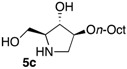	16%	13.7%	14%
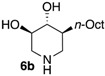	85%	91%	84%
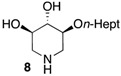	ND	99%	94%

^1^ Cell viability was evaluated using the MTT test as reported earlier [37,38]. Viability of the treated cells is expressed as percentage of that of untreated cells. ^2^ B16 cells, compounds were used at 25 μM. ^3^ Compounds were used at 30 μM. ^4^ 1541F02 pAS cells, compounds were used at 30 μM. ^5^ Not Determined.

**Table 2:** Effect of iminosugars on GCS and GBA inhibition.

Enzyme Inhibition	GCS ^1^	GBA ^2^
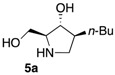	15%	>8000 nM
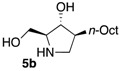	47%	4200 nM
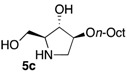	20%	6000 nM
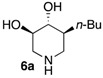	NI ^3^	108 nM
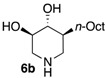	NI	11 nM
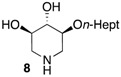	ND ^4^	4 nM

^1^ Evaluation of GCS inhibition was performed as previously reported [37,38]. B16 murine melanoma cells were incubated overnight in the presence or absence of 25 μM of indicated compounds and biosynthesis of GlcCer was then assessed. Data are expressed as a percentage of the values in B16 untreated cells. ^2^ GBA activities were determined at pH = 5.4 using 4-methyl- umbelliferyl-β-d-glucopyranoside as substrate and Cerezyme^TM^ (Sanofi Genzyme) as the enzyme source. Data are expressed as the concentration in tested compound giving 50% of inhibition (IC_50_). ^3^ No inhibition observed. ^4^ Not determined.

## Supplementary Materials

The following are available online. Figure S1: ^1^H and ^13^C-NMR spectra of compound **6b.** Figure S2: ^1^H and ^13^C-NMR spectra of compound **8.** Figures S3–S8: IC_50_ curves for compounds **5a**–**5c** and **6a**, **6b** and **8.**
