# Supplementary material for: Selective Targeting of the Interconversion between Glucosylceramide and Ceramide by Scaffold Tailoring of Iminosugar Inhibitors
Source: Molecules. 2019 Jan 19;24(2):354. doi: 10.3390/molecules24020354 (PMC6359432; doi:10.3390/molecules24020354)
Supplement: Supplementary file 1 [file molecules-24-00354-s001.pdf]

Supplementary Information

Selective targeting of the interconversion between glucosylceramide and ceramide by scaffold tailoring of iminosugar inhibitors

Cécile Baudoin-Dehoux,* Tessa Castellan, Frédéric Rodriguez, Arnaud Rives, Fabien Stauffert, Virginie Garcia, Thierry Levade, Philippe Compain* and Yves Génisson*

List of contents

**Figure S1.**	^{1}H and ^{13}C NMR spectra of compound **6b**--3
**Figure S2**	^{1}H and ^{13}C NMR spectra of compound **8**--4
**Figures S3-S8**	IC_{50} curves for compounds **5a-5c** and **6a**, **6b** and **8**---------------------------------5

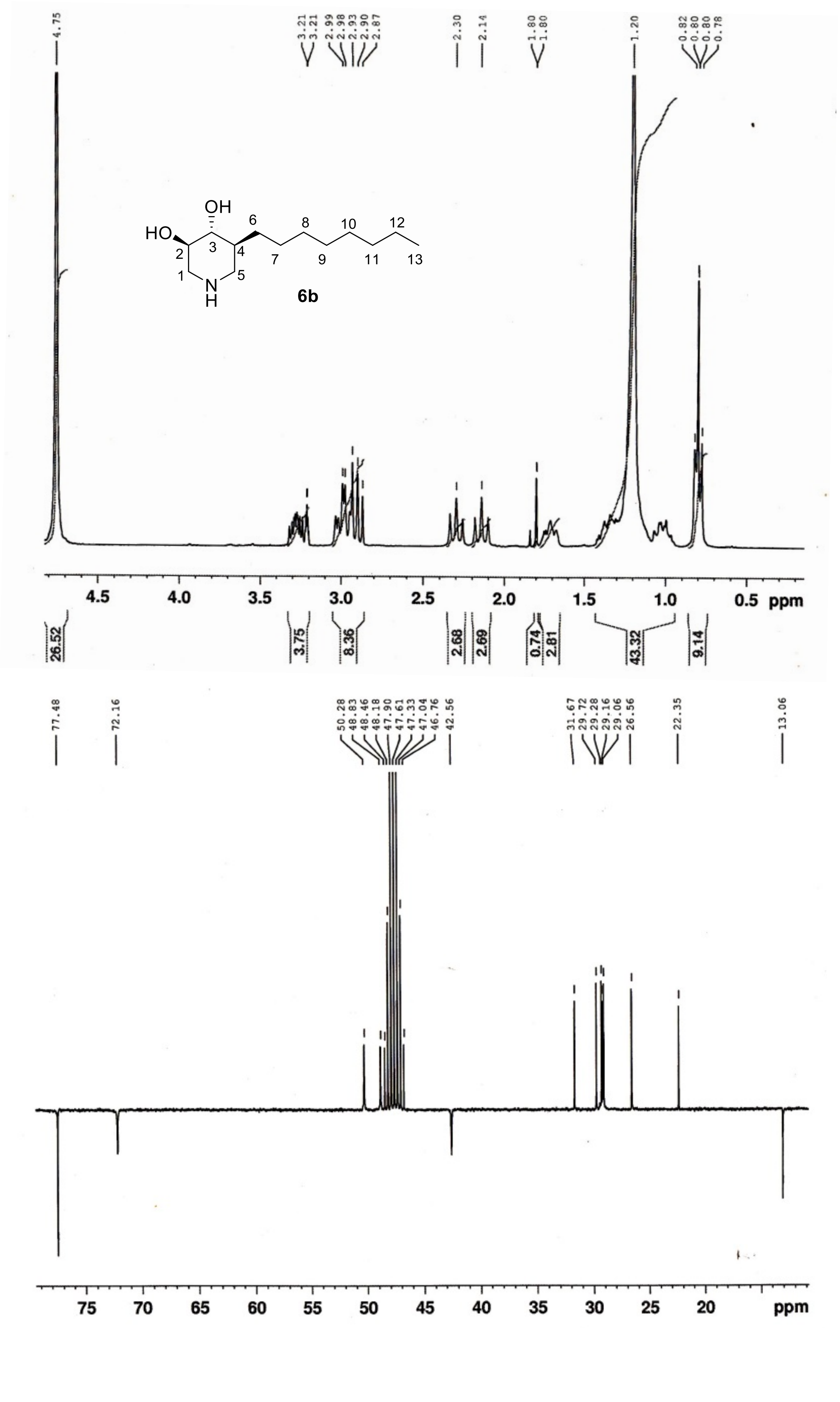

Figure S1. ^{1}H and ^{13}C spectra (300 MHz, 75 MHz, CD_3OD) of **6b**

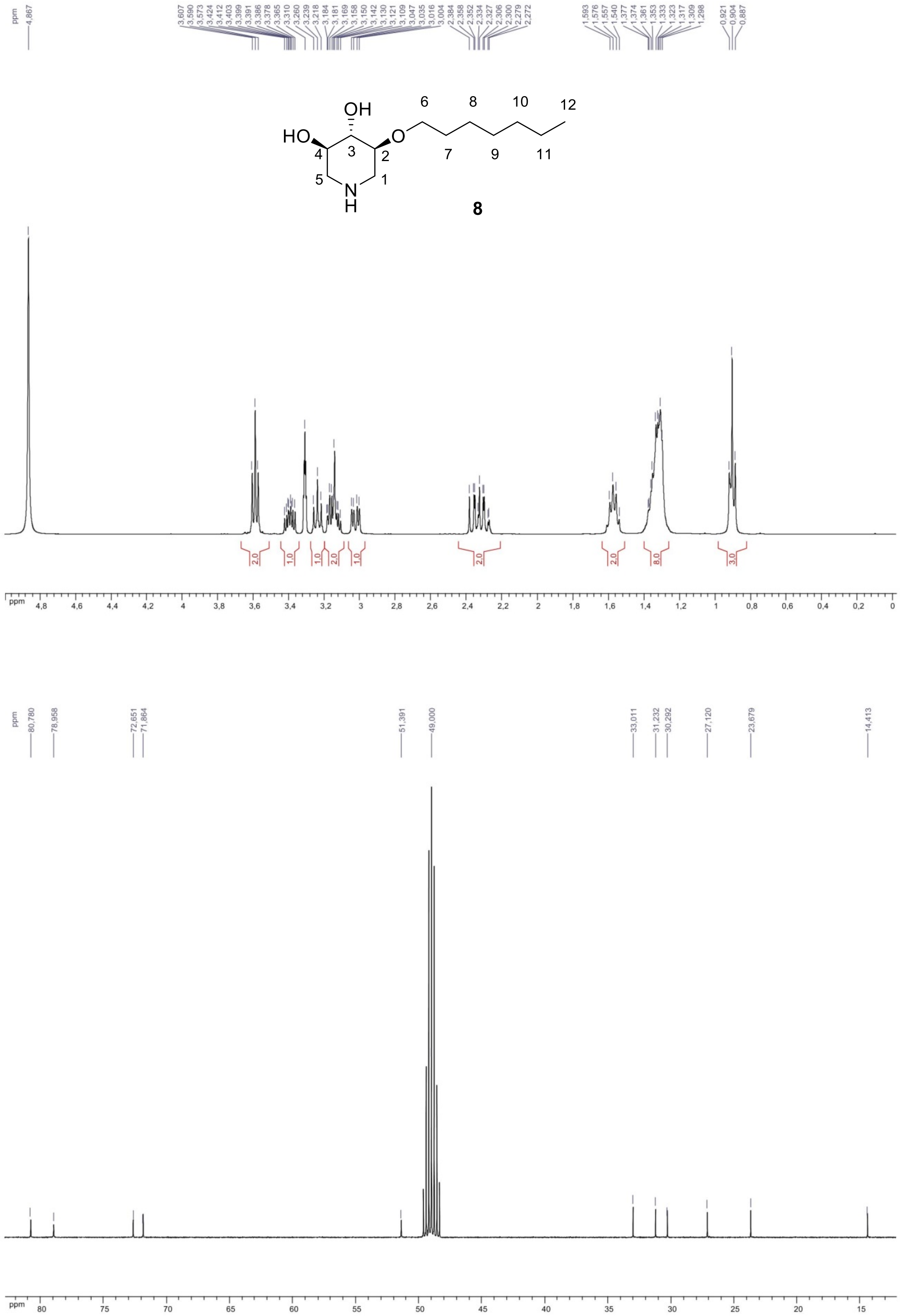

Figure S2. ^{1}H and ^{13}C spectra (300 MHz, 75 MHz, CD_3OD) of **8**

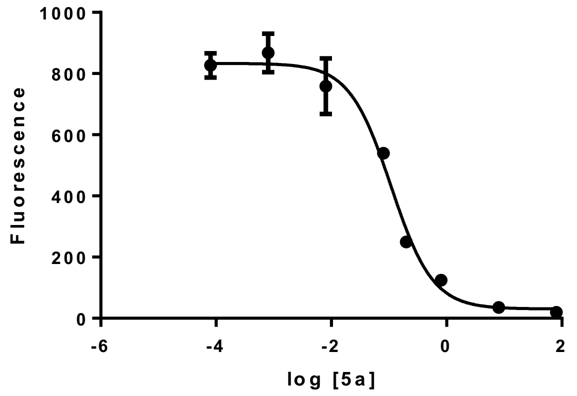

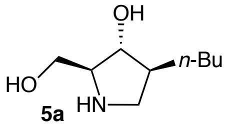

IC_{50} = 8000 nM

Figure S3: dose-response curve of recombinant GBA inhibition by compound 5a at pH 5.4

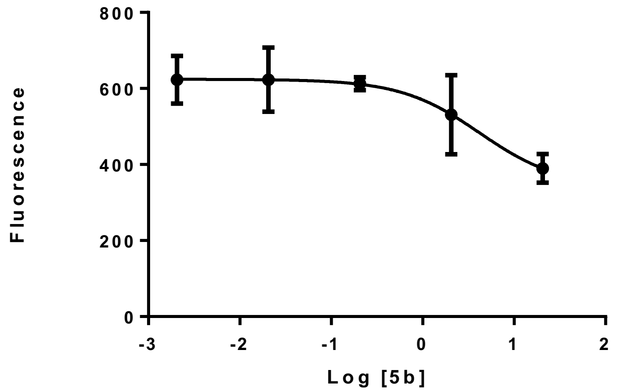

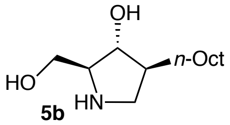

IC_{50} = 4 200 nM

Figure S4: dose-response curve of recombinant GBA inhibition by compound 5b at pH 5.4

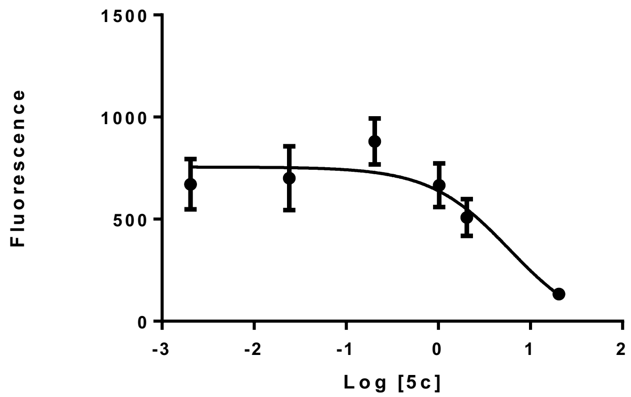

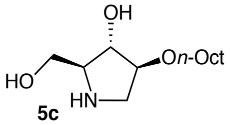

IC_{50} = 6 000 nM

Figure S5: dose-response curve of recombinant GBA inhibition by compound 5c at pH 5.4

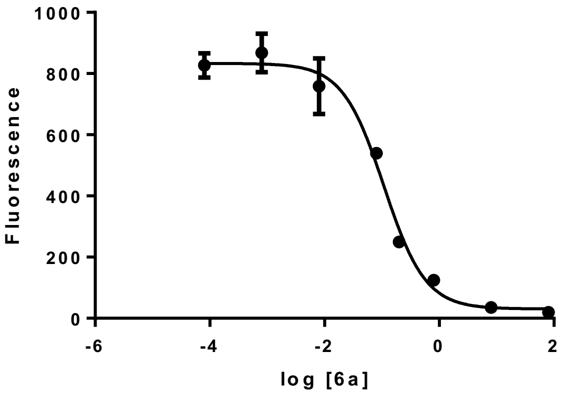

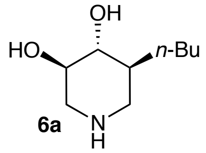

IC_{50} = 108 nM

Figure S6: dose-response curve of recombinant GBA inhibition by compound 6a at pH 5.4

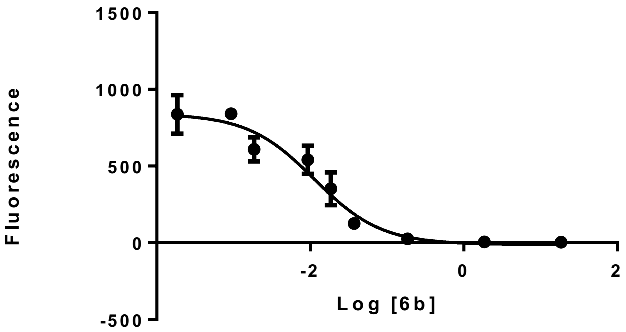

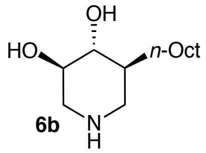

IC_{50} = 11 nM

Figure S7: dose-response curve of recombinant GBA inhibition by compound 6b at pH 5.4

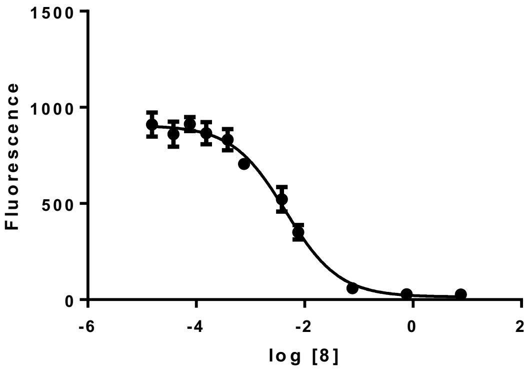

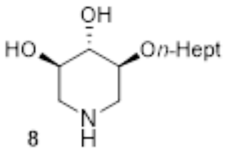

IC_{50} = 4 nM

Figure S8: dose-response curve of recombinant GBA inhibition by compound 8 at pH 5.4
